# Emerging methods for the characterization of ischemic heart disease: ultrafast Doppler angiography, micro-CT, photon-counting CT, novel MRI and PET techniques, and artificial intelligence

**DOI:** 10.1186/s41747-021-00207-3

**Published:** 2021-03-25

**Authors:** Martin J. Willemink, Akos Varga-Szemes, U. Joseph Schoepf, Marina Codari, Koen Nieman, Dominik Fleischmann, Domenico Mastrodicasa

**Affiliations:** 1 Department of Radiology, Stanford University School of Medicine, 300 Pasteur Drive, Stanford, CA 94035 USA; 2 Division of Cardiovascular Imaging, Department of Radiology and Radiological Science, Medical University of South Carolina, Charleston, SC USA; 3 Division of Cardiovascular Medicine, Stanford University School of Medicine, Stanford, CA USA; 4 Stanford Cardiovascular Institute, Stanford, CA 94305 USA

**Keywords:** Artificial intelligence, Coronary artery disease, Myocardial infarction, Myocardial ischemia, Radiology

## Abstract

After an ischemic event, disruptive changes in the healthy myocardium may gradually develop and may ultimately turn into fibrotic scar. While these structural changes have been described by conventional imaging modalities mostly on a macroscopic scale—*i.e*., late gadolinium enhancement at magnetic resonance imaging (MRI)—in recent years, novel imaging methods have shown the potential to unveil an even more detailed picture of the postischemic myocardial phenomena. These new methods may bring advances in the understanding of ischemic heart disease with potential major changes in the current clinical practice. In this review article, we provide an overview of the emerging methods for the non-invasive characterization of ischemic heart disease, including coronary ultrafast Doppler angiography, photon-counting computed tomography (CT), micro-CT (for preclinical studies), low-field and ultrahigh-field MRI, and ^11^C-methionine positron emission tomography. In addition, we discuss new opportunities brought by artificial intelligence, while addressing promising future scenarios and the challenges for the application of artificial intelligence in the field of cardiac imaging.

## Key points

- Coronary ultrafast Doppler angiography detects the abnormal flow of postischemic intramural myocardial vessels.
- Photon-counting computed tomography (CT) allows for reduced radiation dose, increased spatial resolution, and differentiation between multiple contrast agents.
- Micro-CT combines functional cardiac imaging with myocardial metabolic assessment.
- Cardiac imaging has become feasible at low and ultrahigh-field MRI.
- ^11^C-methionine positron emission tomography is used to characterize myocardial postinfarction inflammation.

## Background

Ischemic heart disease (IHD) is the leading cause of death globally [1, 2]. The assessment of myocardial ischemic changes is crucial to diagnose IHD accurately, to estimate patients’ prognosis, and therefore to evaluate optimal therapeutic options [3]. Traditionally, stress echocardiography and invasive angiography have been valuable means for IHD diagnosis and treatment, respectively [4–6]. Cardiovascular computed tomography (CT) and magnetic resonance imaging (MRI) provide sophisticated and complementary information through an exceptional high-quality visualization of coronary arteries, as well as of the cardiac structures and function. In addition, nuclear medicine imaging is a well-established tool for the non-invasive assessment of myocardial perfusion in patients with suspected IHD. Extensive research has described the strengths and weaknesses of these conventional imaging modalities [7–9]. Recently, novel imaging methods have shown the potential to unveil a highly detailed picture of the postischemic myocardial phenomena, which may lead to a more comprehensive evaluation of IHD [10, 11]. There is evidence suggesting these new methods may bring pivotal advances in the understanding of IHD, with potential major changes in the current clinical practice.

**Fig. 1:**
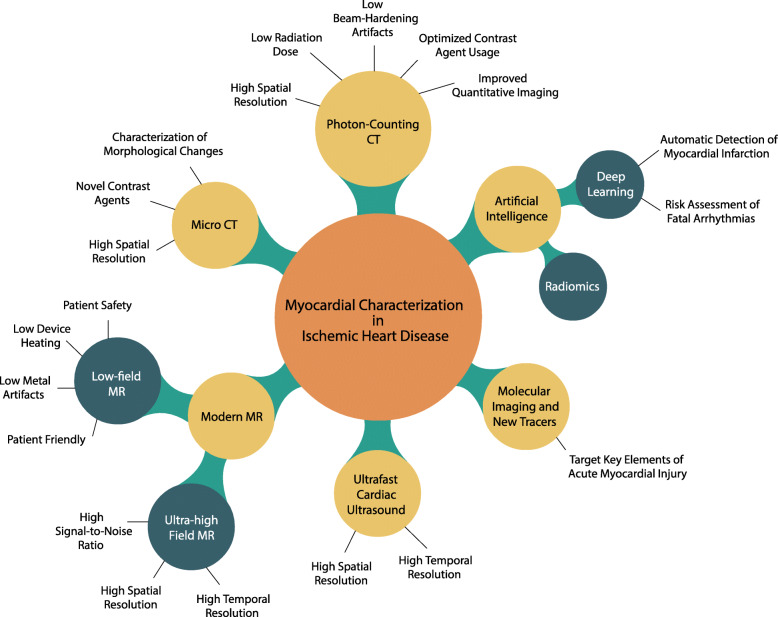
Overview of the emerging methods and tools for the noninvasive characterization of the ischemic myocardial tissue

In this review article, we provide an overview of the emerging methods and tools for the noninvasive characterization of the ischemic myocardial tissue (Fig. 1). We considered techniques used in both the clinical and preclinical setting, including ultrafast cardiac ultrasound, x-ray-based technologies (micro-CT and photon-counting CT), molecular imaging, and low- and ultrahigh-field MRI. Finally, we provide a brief update on the most recent applications of artificial intelligence (AI) in the field of cardiac imaging.

## Ultrafast ultrasound

**Fig. 2:**
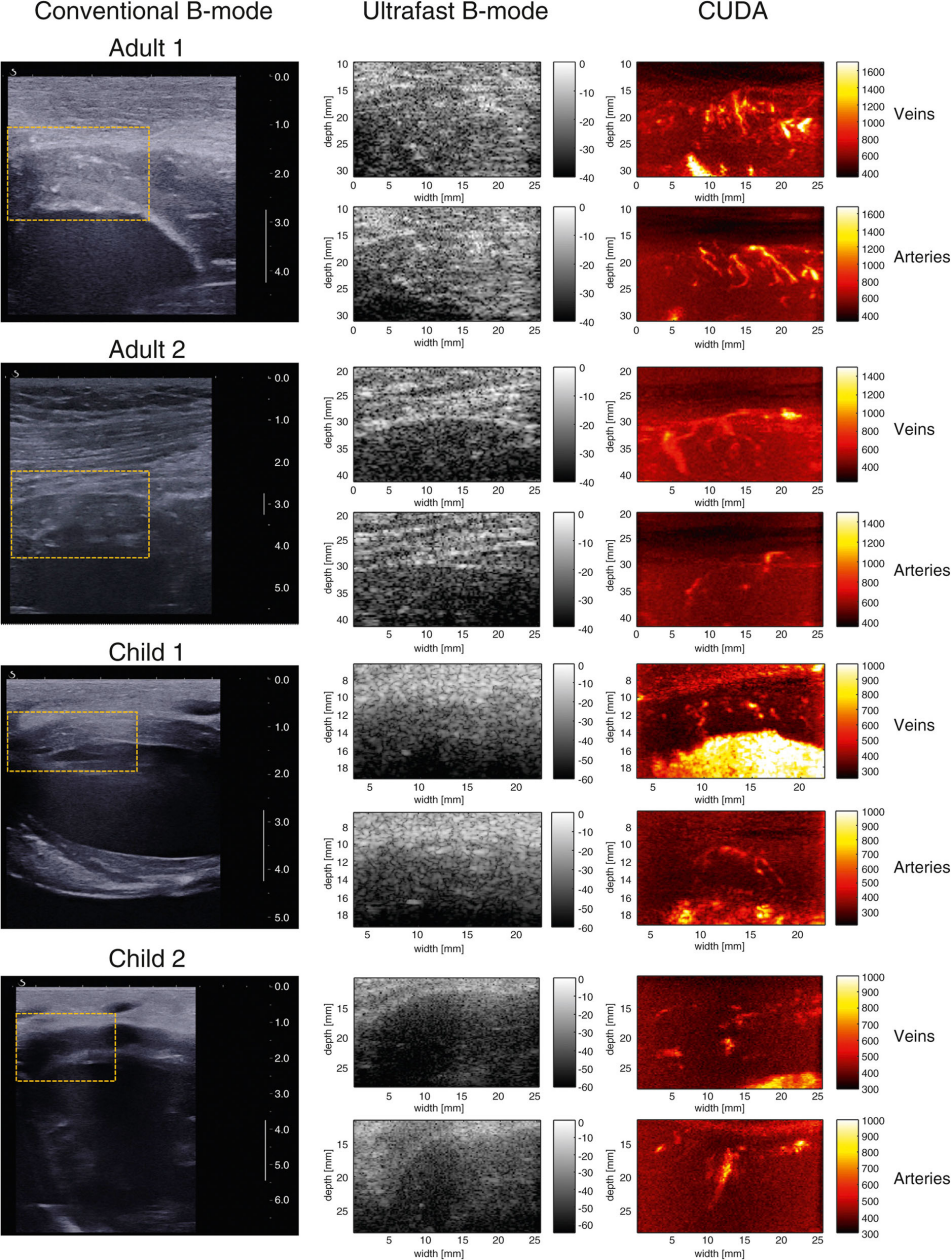
Left: conventional ultrasound image showing the region of interest selected for coronary ultrafast Doppler angiography (CUDA) processing. This real-time imaging mode of the ultrasound scanner was used for positioning (the scale bar is in centimeters). Middle: ultrafast B-mode images of the region of interest depicted in yellow on the images in the left column. Right: CUDA images of coronary veins in systole and coronary arteries in diastole. (Reprinted, with permission, from [10])

Coronary microvascular dysfunction is considered an important prognostic marker for myocardial ischemia [12, 13]. However, the *in vivo* assessment of the human coronary tree has been limited mostly to the epicardial vasculature, thus leaving the intramural vessels largely unexplored. The most recent technological advancements in echocardiographic imaging allow to image the heart at a pace 100 times faster than conventional echocardiography, with higher spatial and temporal resolution [14]. This novel imaging approach, known as ultrafast cardiac ultrasound, has been tested in the preclinical arena and has shown the potential to unveil new information in areas of myocardial mechanics and vascular flow analysis mostly unexplored so far. Recently, Maresca et al. [10] reported the use of cardiac ultrafast ultrasound in combination with adaptive coronary Doppler processing to visualize epicardial and intramural coronary vessels. The authors conducted a series of open-chest experiments on nine swine models using a new technique they named coronary ultrafast Doppler angiography (CUDA). In their experiment, the authors determined coronary flow changes after a brief (30 s) occlusion of the left anterior descending artery followed by reperfusion, to study the hyperemia occurring in the anterior wall of the left ventricle. In addition, the authors obtained noninvasively the coronary flow reserve after the intravenous administration of adenosine. A left anterior descending artery flowmeter probe was used to validate their findings. Later, the left anterior descending artery occlusion was prolonged to 90 min to induce myocardial infarction and documented abnormal arterial and venous flow. Interestingly, in an effort to translate this technology into clinical practice, CUDA was initially tested on four healthy human volunteers, two adults and two pediatric patients, only for the visualization of the coronary vasculature (Fig. 2). Both arterial and venous intramural vessels were detected in a beating heart, though at the cost of a lower image spatial resolution due to the traditional transthoracic approach.

As radiation and contrast-free imaging modality, CUDA is a promising tool in the field of myocardial injury characterization. More so, if we consider its ability to assess coronary flow reserve noninvasively, which would be paramount in patients with stable coronary disease. However, there are still critical hurdles to its clinical implementation. CUDA is based on the use of a linear transducer array that extends the imaging depth to a maximum of 45 mm, a distance that is unfit for the majority of adult patients. Moreover, CUDA was unable to provide an absolute quantification of the flow rate. Nonetheless, CUDA provided a solid foundation for *in vivo* imaging of intramural coronary vessels and coronary blood flow variations.

## Micro-CT

**Fig. 3:**
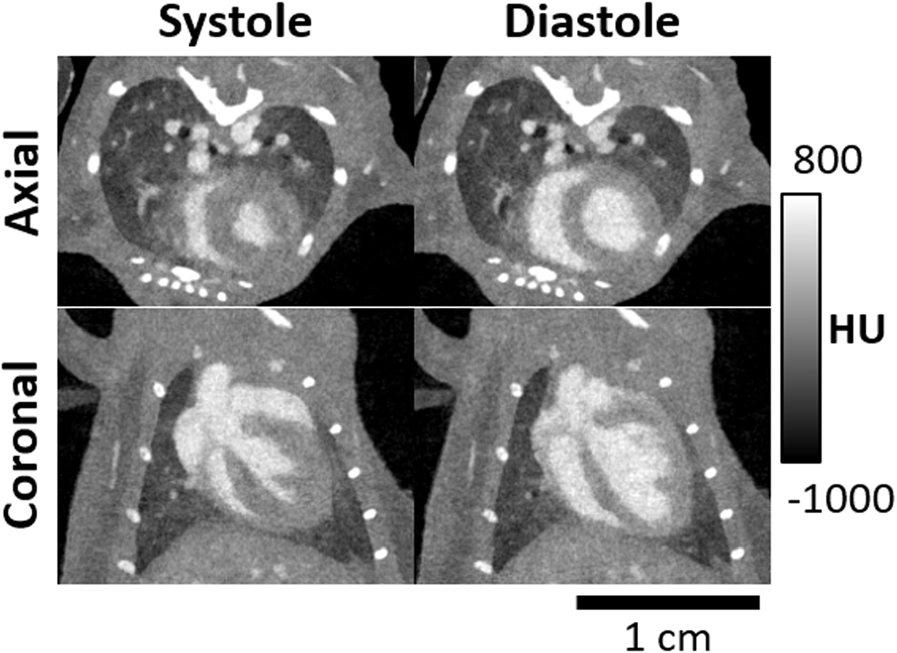
Axial and coronal views of systole and end-diastole in a mouse model with intravascular iodine contrast. Projection data were sampled using a prospective gating scheme. These images represent two of the ten cardiac acquired phases, *i.e.*, diastole and systole. Reconstruction was performed using a four-dimensional iterative technique based on the split Bregman method [16]. Figure courtesy of Dr. Cristian T. Badea, PhD, Duke University

Micro-CT is a well-established preclinical research tool, because of its high spatial resolution and the opportunity to characterize morphological changes in anatomical structures approaching the micrometer range. Moreover, modern micro-CT scanners are equipped with advanced cardiorespiratory gating and are able to acquire four-dimensional datasets [15], therefore providing an accurate assessment of the cardiac function (Fig. 3). Both *in vivo* and *ex vivo* small animal studies have been used as models for human cardiovascular conditions, ranging from atherosclerosis [17–21] to congenital heart defects [22, 23] to enhance the understanding of diseases and develop potential targeted therapies. Sangaralingham et al. [22] investigated the aging coronary vasculature in Fischer rats. They found that micro-CT was able to detect the reduction in intramyocardial vessel volume in the aged heart along with increased epicardial vessel volume, left ventricular fibrosis, and mild dysfunction. Pai et al. [19] demonstrated that micro-CT could also be used for vessel wall imaging of the coronary arteries in mice. The authors used osmium tetraoxide as a tissue-staining contrast agent that is retained in the vessel wall and reported that coronary arteries as small as 45 μm in diameter can be visualized. While micro-CT has been proven to be able to acquire isotropic data on cardiac morphology and function, it also has the advantage of being faster than MRI, therefore providing high-resolution image data, even under dobutamine-induced stress, as demonstrated by Badea et al. in healthy adult rats [24].

Due to the increased spatial resolution of micro-CT and consequent possibility to visualize microstructures and metabolic processes, novel contrast agents had to be developed to provide tissue differentiation on the microscopic level. Ashton et al. [25] investigated the preclinical use of eXIA 160, an aqueous colloidal polydisperse contrast agent with a high iodine concentration taken up by the myocardium and other metabolically active tissues. More specifically, they demonstrated the potential role of eXIA 160 for imaging myocardial infarction in mice: immediately after eXIA 160 intravenous administration, an about 340 HU signal density difference between blood and myocardium allowed for ventricular volumetry and function assessment. In addition, the about 140 HU contrast difference between normal and infarcted myocardium four hours after the injection enabled quantification of infarct size using dual-energy micro-CT, validated against micro-single-photon emission computed tomography and *ex vivo* histopathology. Van Deel et al. [26] demonstrated similar results using the same contrast agent in micro-CT studies for the assessment of global and regional myocardial function, myocardial perfusion, metabolism, and viability in a mouse model of myocardial ischemia induced by permanent occlusion of the left anterior descending coronary artery.

Among the other innovative contrast agents, ExiTron MyoC 8000, a nanoparticulate agent, was optimized for CT studies of the myocardium. It is composed of a polymer shell and a liquid core, with a mean hydrodynamic diameter of about 300 nm, showing ultrahigh density values (about 8,000 UH). Its accumulation in the healthy myocardial tissue combine with its long blood half-life (about 2 h in mice) allows for visualizing myocardial pathological changes as well to perform functional cardiac imaging [27]. This agent has been tested in micro-CT to longitudinally monitor cardiac processes *in vivo,* in mice with myocardial infarction [28]. ExiTron MyoC 8000-enhanced micro-CT imaging was able to detect and accurately quantify myocardial infarction, even at a very low contrast dose (50 μL per 25-g mouse body weight, 210 mg I/mL).

The main limitation of micro-CT is the size of the CT tube that is able to accommodate small animals only. While less relevant to animal studies, the substantial radiation exposure will pose further challenges when human studies will be considered.

## Photon-counting CT

CT is a remarkably successful technology, which is reflected in the increasing number of exams performed annually [27]. Despite its success, CT has important limitations including (1) relatively high x-ray radiation exposure, (2) limited soft tissue differentiation, and (3) use of iodinated contrast agents [11]. Some of these issues can theoretically be solved by dual-energy CT, which provides iodine concentration maps and material-specific images. Separation between high-energy and low-energy x-ray photons (the so-called spectral separation) is, however, suboptimal with current dual-energy CT systems [29]. A more robust solution that has the potential to address current technical CT limitations is photon-counting CT. Despite the fact that photon-counting CT is currently not yet commercially available, it is expected that this technique will dramatically change CT imaging [11].

The improved noise behavior of photon-counting detectors may allow for reduced radiation exposure. This is relevant for cardiovascular imaging since, despite major improvements over the last decades, radiation doses are still substantial for cardiovascular imaging protocols [30]. Symons et al. [31] assessed the performance of photon-counting CT for coronary artery calcium quantification in 10 *ex vivo* hearts and 10 asymptomatic volunteers without electrocardiography gating. Their results indicated that either image quality of coronary artery calcium scans can be achieved or radiation dose can be reduced with photon-counting CT. Kappler et al. [32] assessed water phantoms and found increased attenuation of iodine with comparable image noise as conventional energy-integrating detectors. The increased contrast-to-noise ratios may allow for a reduction in radiation dose of up to 32%, as confirmed by recent investigations conducted in the field of brain [33] and chest imaging [34].

Smaller detector elements typical of photon-counting CT scanners may allow for improved spatial resolution. Currently, assessment of coronary stenoses is substantially limited in the presence of calcium or stents due to blooming artifacts. Since blooming is dependent on the point-spread function of the CT system, the increased spatial resolution of photon-counting detectors may result in less blooming [35]. Multiple recent studies have evaluated the utility of high spatial resolution photon-counting CT for cardiovascular applications. Symons et al. [36] conducted a phantom study with 18 coronary stents with diameters ranging from 2.0 to 4.0 mm and found significantly improved visibility of the coronary stent lumen. Similarly, Mannil et al. [37] assessed 18 coronary stents with a diameter of 3.0 mm with luminal iodinated contrast and found superior in-stent lumen delineation compared with conventional energy-integrating detectors.

Beam-hardening artifacts can be corrected for due to improved material decomposition. Beam-hardening artifacts caused by surrounding anatomical structures or implanted devices hamper the evaluation of myocardial perfusion. Decreased beam-hardening artifacts may thus enhance the performance of myocardial perfusion CT. Symons et al. [38] acquired photon-counting CT angiography exams of the head and neck region in 16 volunteers with a 140-kV tube voltage. All volunteers also underwent a 120-kV exam with a conventional CT system at a comparable radiation dose. Despite the higher tube voltage, contrast-to-noise ratios were similar, and most importantly, beam-hardening artifacts were less severe in the photon-counting CT exams. Using the high-energy portion of photon-counting detector data can also reduce beam-hardening artifacts. A study conducted by Pourmorteza et al. [33] in 21 asymptomatic volunteers suggested that high-energy photons were less affected by beam hardening. The disadvantage is that this approach results in reduced tissue contrast.

The use of contrast agents may be optimized with photon-counting CT. Patients who are allergic to iodine or patients with decreased kidney function would benefit from CT image acquisition with reduced iodine concentrations or other contrast agents, such as gadolinium. Since photon-counting CT has the potential to improve contrast-to-noise ratio, not only radiation dose can potentially be decreased, also iodine contrast concentrations may be reduced. A variety of studies has shown improved contrast-to-noise ratio with photon-counting CT compared with conventional energy-integrating detectors. Yu et al. [39] showed in a phantom study that the contrast-to-noise ratio of iodine *versus* water increased by up to 25.5%, which may allow for iodine contrast load reduction. Besides iodine load reduction, alternative contrast agents, either through systemic or targeted administration, may also be considered with photon-counting CT, such as gadolinium, barium, gold, and platinum. Cormode et al. [40] scanned apolipoprotein E knockout mice on a photon-counting CT system after injecting gold nanoparticles and iodine. They concluded that valuable information might be yielded about atherosclerotic plaque composition. This indicates that photon-counting CT allows for differentiation between simultaneously administered multiple contrast agents, and the specific distribution of these contrast agents may result in additional information. Symons et al. [41] intravenously administered gadolinium and iodine and orally administered bismuth in an occlusion-reperfusion canine model of myocardial infarction. This study showed that concentrations of these contrast agents, as well as the wash-in wash-out kinetics, could be quantified.

Photon-counting CT offers the opportunity to improve quantitative imaging compared with current CT technology. Materials and tissues can be differentiated with color-coded images that are material/tissue specific. Not only can different materials be displayed, but also concentrations of materials, such as contrast agents and calcium, can be quantified, independent of acquisition settings. Quantification of material concentration may benefit myocardial perfusion analyses since this allows for the exact concentration determination of contrast agents in the myocardium. Moreover, coronary calcium is currently quantified with the Agatston score [42], which is dependent on acquisition parameters such as tube voltage and reconstruction parameters such as slice thickness. Quantification of calcium with photon-counting CT is independent of these parameters and may thus allow for the exact quantification of coronary calcium mass. Another advantage may be a more precise quantification due to reduced blooming artifacts.

Unfortunately, the ideal photon-counting CT system does not exist yet. Currently, only prototype systems are in development for human-sized imaging. It is expected that the first commercial photon-counting CT scanners for humans will become available within the next 3 to 10 years. Before clinical implementation, important challenges need to be tackled. *Cross-talk* is still a problem with current photon-counting detectors [43]. Despite reduced cross-talk compared with conventional energy-integrating detectors, x-ray photons may hit the detector right on the border between two detector elements. Also, part of the energy may be released in the form of fluorescence photons, which may move to a random location, potentially a neighboring detector element, resulting in cross talk. Many incoming x-ray photons hit CT detectors in a short time [44]. To be able to count every photon separately, a fast detector is required. Pileup of x-ray photons is currently still a problem with photon-counting detectors. Since x-ray photon count rates are lower in micro-CT imaging, photon-counting detectors are also evaluated for micro-CT [45]. Up to our knowledge, there are currently no photon-counting CT systems available that allow for electrocardiography synchronization, which is necessary for myocardial infarction assessment of CT images. However, it is expected that electrocardiography synchronization will be enabled on photon-counting CT in the short term.

## Molecular imaging and new tracers

Myocardial infarction and the subsequent reperfusion of the infarcted myocardial tissue ignite a complex series of biological events culminating in the development of a fibrotic scar [46]. The detailed characterization of these phenomena represents a challenging task for imaging modalities commonly used in clinical practice since these techniques currently tend to focus more on the macroscopic anatomical and functional aspects of the heart. In the last few decades, new positron emission tomography (PET) tracers and MRI agents have been developed to target key elements of acute myocardial injury, defined by cardiomyocyte death and endothelial damage, and chronic cardiac remodeling. For instance, Sosnovik et al. [47] targeted Annexin-V, a marker expressed in apoptotic cells, to characterize acute ischemia and heart failure in mice. Imaging targets sensitive to vascular permeability have been tested as well. A family of cell-surface proteins known as integrins are considered a marker of angiogenic vascular tissue. Α_v_β_3_ is an integrin whose expression is abundant in newly formed blood vessels, which is a process associated with endothelial damage [48]. Arginyl-glycyl-aspartic acid (RGD) is a short peptide sequence recognized by integrins, and it is expressed on extracellular matrix proteins and membrane surfaces. Several preclinical and clinical studies showed that RGD-based radiotracers labeled with fluorine and gallium were able to document the angiogenesis occurring in the regeneration process following myocardial infarction [49, 50]. Notably, only ^18^F-galacto-RGD has been described in patients with myocardial infarction [49]. Recently, Lavin et al. [51] developed a new gadolinium-based albumin-binding contrast agent, gadofosveset, and demonstrated the feasibility of imaging vascular permeability in a murine model of myocardial infarction.

Another key process of the postinfarction cardiac remodeling is the deposition of new extracellular matrix, typically rich in collagen and elastin. Elastin can preserve elasticity in the infarcted regions, thus leading to an improved ejection fraction [52]. As such, elastin represents an interesting remodeling biomarker, along with its precursor tropoelastin. Elastin/tropoelastin-specific MRI contrast agents have been used to assess the extracellular matrix remodeling in preclinical studies [53–55].

Monocytes and leukocytes are among the several immune cells generating an inflammatory response within the ischemic myocardium. These cells have been targeted to characterize the myocardial inflammation occurring after an ischemic insult. For instance, ^18^F-fluorodeoxyglucose (FDG), a tracer well-known in the field of cancer imaging, has been used in association with PET-CT to characterize the metabolic activity of the infarcted myocardium. The infarcted myocardium is, in fact, rich in leukocytes [56], which have an elevated metabolic activity. However, the FDG uptake by viable cardiac cells represents a confounding factor. To suppress the signal from normal cardiac cells, patients have to follow specific dietary restrictions [57]. The results tend to be suboptimal, and therefore, more specific tracers are warranted. ^18^F-fluorodeoxymannose is a potential alternative, based on a glucose isomer (mannose), suggested for imaging of macrophages [58]. So far, its applications have been limited to preclinical studies.

Macrophages have also been targeted using ^68^Ga-DOTATATE and ^68^Ga-DOTATOC, two radiotracers with an affinity for the somatostatin receptors types 2 and 5, respectively, expressed in macrophages [57–60]. ^68^Ga-pentixafor is a PET radiotracer targeting the chemokine receptor CXCR4, which is thought to play a role in leukocyte recruitment to the injured myocardial tissue. ^68^Ga-pentixafor has been used in mice and translated to patients to highlight the expression of CXCR4 during the early inflammatory state occurring after myocardial infarction [61, 62]. Interestingly, the expression of CXCR4 was more heterogeneous in patients, suggesting a unique and complex clinical environment for each patient, which could have important implications in patients’ prognosis and therapy.

^11^C-methionine is a clinically approved agent, known mostly in the oncologic field, which accumulates in macrophages [63]. Therefore, it represents an ideal imaging target to characterize postinfarction myocardial inflammation. A PET study using ^11^C-methionine demonstrated a high signal in the early stages of myocardial infarction, declining seven days from the ischemic insult [64]. The peculiar temporal window of ^11^C-methionine indicates an accurate characterization of the early stages of myocardial infarction and, therefore, with important implications for the assessment and efficacy of targeted therapies.

Although most of the above-described studies are preclinical, they are immensely relevant because they showed the potential to provide more detailed information on IHD, therefore leading to a more tailored patient management and optimized therapies. More research is needed to validate these new options in large clinical populations.

## Low-field and ultrahigh-field MRI

Cardiac MRI has the advantage to provide high soft tissue contrast and therefore differentiation of various tissue entities within the myocardium. Moreover, it has the ability to evaluate function, perfusion, and flow in addition to structure. While the standard MRI field most commonly used for cardiac applications is 1.5 T, there are certain benefits, and also shortcomings, of moving towards the lower or upper end of the currently achievable magnetic field range as detailed below.

Low-field MRI (≤ 0.35 T) has been around for decades and we now see the revamp in the utilization of this technique again due to technology improvements allowing for cardiac imaging at such a low-field range. As recently demonstrated by Simonetti and Ahmad [65], low-field MRI has the advantage of improved patient safety (low ferromagnetic attraction, practically no specific absorption rate limits), reduced device heating and artifacts related to metallic implants, reduced equipment and maintenance cost, and more patient-friendly design since the bore of the low-field magnets can be configured as open MRI systems. Such low-field applications may be beneficial in patients with *de novo* implants when MRI is usually not recommended during the first two weeks. While low-field MRI comes with the disadvantage of low signal-to-noise ratio, Simonetti and Ahmad reported promising results with cine, phase-contrast, and black blood cardiac imaging, using advanced image reconstruction techniques, such as Sparsity Adaptive Compressive Recovery (SCoRe) [65], which allows for adequate image quality to generate diagnostic images. The further potential advantages of low-field MRI are the lower equipment and operational costs and the improved simplicity of image acquisition.

**Fig. 4:**
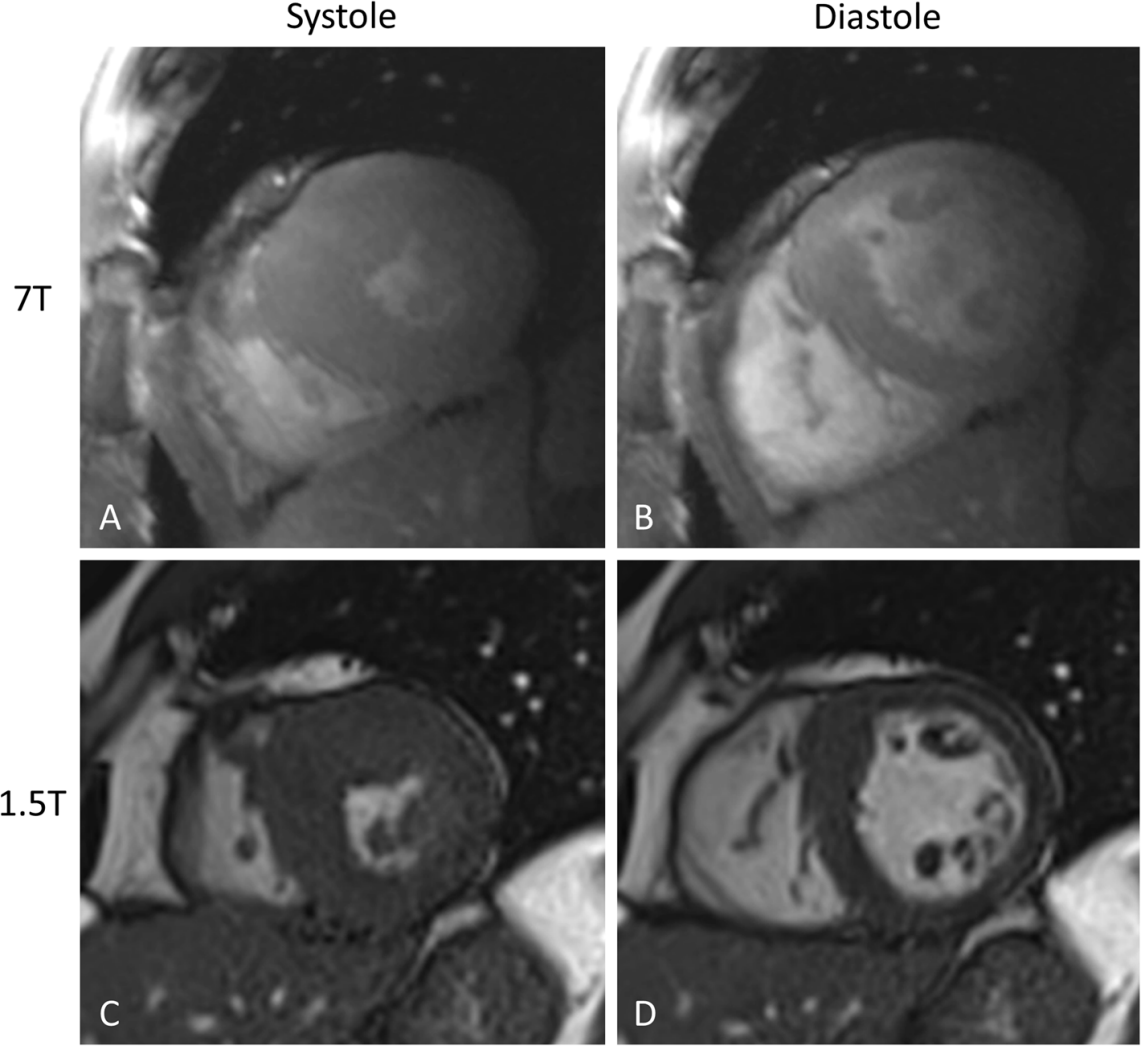
Systolic and diastolic cine images acquired at 7 T (**A,** **B**) and 1.5 T (**C,** **D**) in the same subject. Despite the challenges with electrocardiography interference and triggering, diagnostic quality cine images can be acquired with high temporal and spatial resolution. Figure courtesy of Dr. Harold I. Litt, University of Pennsylvania

As MRI technology advances towards the direction of higher field strengths as seen recently by the increasing number of 7-T whole body systems installed in multiple academic institutions, it is important to assess what advantages can be gained for IHD assessment by such high field and what drawbacks we are facing. A clear benefit of 7-T systems is the exceptional signal-to-noise ratio as well as increase in spatial and temporal resolution [66]. The limitations, however, are substantial, especially for cardiovascular imaging [67, 68]: increased heating of metal implants, increased ferromagnetic attraction, increased field inhomogeneity, more prominent electrocardiography interference caused by magneto-hydrodynamic effects [69] and increased metallic device-related artifacts [65]. A 7-T image example is shown in Fig. 4.

Despite these limitations, recent literature demonstrates that the use of 7-T systems, either as small bore for experimental research or large bore for human applications, has potential in certain fields of cardiovascular imaging. Zhang et al. [70] investigated and found T2 mapping feasible and reliable at 7 T to characterize myocardium in order to quantify the area at risk, infarct core, intramyocardial hemorrhage, and salvaged myocardial zone in a rat model of myocardial infarction. Spath et al. [71] tested the application of manganese-enhanced MRI in a rat model of myocardial infarction. They found that manganese-enhanced MRI causes calcium channel-dependent myocardial T1 shortening, and T1 mapping was able to provide an accurate assessment of infarct size and changes in calcium handling in the remote myocardium, which may hold potential for the assessment of myocardial viability, remodeling, and regeneration. Myocardial infarction in a rat model has also been studied using Gadofluorine P in a combined MRI and mass spectrometry approach [72]. Wang et al. [73] demonstrated that late gadolinium enhancement imaging can be performed at 7 T in a rat model of myocardial infarction in a self-gated fashion, *i.e.,* without electrocardiography and respiratory gating.

Promising approaches have been proposed for 7-T human cardiac imaging as well. An acoustic cardiac triggering technique has been introduced by Frauenrath et al. [74] for synchronization and gating of cardiac MRI studies at 7 T, which has been demonstrated to have superior reliability and fidelity compared with that of conventional vector electrocardiography and traditional pulse oximetry. Acoustic cardiac gating is based on the phonocardiogram signal, and uses the first heart tone for acquisition triggering. von Knobelsdorff-Brenkenhoff et al. [75] studied fast gradient-echo imaging for cardiac chamber volume quantification at 7 T in nine subjects, and found it feasible and in good agreement with balanced steady-state free precession-based measurements in the same subjects at 1.5 T. Hezel et al. [76] investigated the feasibility of human myocardial mapping at 7-T ultrahigh field and found that challenges, such as signal voids, can be addressed by using tailored shimming techniques and dedicated acquisition schemes. Prothmann et al. [77] performed late gadolinium enhancement imaging in 131 patients at 7 T and found the technique feasible to detect myocardial fibrosis with higher spatial resolution compared to 3 T.

While most of these studies aimed to establish feasible techniques for 7-T cardiac imaging, an increasing effort in the scientific community to explore the potentials of ultrahigh-field MRI can clearly be recognized.

Overall, new MRI techniques may potentially improve access to MRI due to the lower cost and improved patient compliance at low field, and open up new avenues in the characterization of the ischemic myocardium by employing the single-to-advantages of high-field imaging.

## AI applications in cardiac imaging

In light of the advent of *big data*, along with the availability of powerful computational technology, AI applications are thriving in the field of medical imaging at an unprecedented pace [78, 79]. This holds true for cardiac imaging, where recent studies have shown preliminary, but promising results that may take cardiac imaging further than ever. Among AI algorithms, deep learning (DL) has been successfully applied, ranging from image reconstruction and denoising, to image classification, segmentation, and interpretation [80–83].

Since “upstream” AI applications, such as image reconstruction and denoising, are more general and not specifically related to IHD diagnosis, in this review, we will focus on “downstream” AI applications specifically related to the topic at issue [84].

For instance, Zreik et al. [85] identified patients with hemodynamically significant coronary stenoses relying solely on the automatic analysis of the left ventricular myocardium on rest coronary CT angiography (CCTA) scans. To this end, the authors used a convolutional neural network to segment the left ventricle myocardium, an unsupervised convolutional autoencoder to extract myocardial features and machine learning classifier to analyze CCTA data. The proposed pipeline reached an average area under the curve (AUC) at receiving operator characteristic analysis of 0.74 ± 0.02 (mean ± SD) during10-fold cross-validation performed in 126 scans. This method was then applied by van Hamersvelt et al. [86] in 101 patients with intermediate coronary stenosis to evaluate the impact of an additional DL-based analysis of the myocardium to identify those with functionally significant stenosis. They reported an improvement in AUC values when the DL approach was combined with the determination of the coronary degree of stenosis against the coronary degree of stenosis alone (0.76 *versus* 0.68). Moreover, though the DL analyses were focused on the myocardium only, the visual assessment of coronary arteries improved also the sensitivity. These studies further confirmed the potential of CCTA as the ultimate gatekeeper for unnecessary invasive angiography studies. Future automated approaches will probably benefit from a more complete evaluation coupling the left ventricular myocardium analysis with computational fluid dynamics applied to the coronary tree.

By using a recurrent neural network, Xu et al. [87] were able to automatically detect areas of myocardial infarction on cardiac MRI scans with an overall pixel classification accuracy of 94.4% in a sample of 114 subjects. This end-to-end DL framework enabled the accurate detection of the infarction size at the pixel level, which has the potential to reduce the inter-observer variability of the visual assessment of late gadolinium enhancement and therefore improve the efficiency in myocardial infarction diagnosis even in less experienced readers.

When LGE imaging cannot be performed due to contraindications to gadolinium-based contrast agents, a non-contrast approach would be highly desirable. Zhang et al. [88] introduced a DL framework to diagnose chronic myocardial infarction in 212 patients using unenhanced cardiac cine MRI images. Their approach allowed to identify chronic myocardial infarction in testing data with sensitivity of 0.90 a specificity of 0.99, and an AUC of 0.94. Based on extracted motion features, the authors proved that it is possible to determine the presence, location, size, and degree of transmural extent of the myocardial infarction without the need for gadolinium-based contrast agent injection.

Radiomics is another promising approach with the potential to expand our knowledge of information contained in cardiovascular digital images and to realize a step forward toward precision medicine [89]. Radiomics is essentially a process enabling the extraction of a large number of handcrafted or DL-based quantitative features from selected areas in radiological images. These features could also be used to identify meaningful associations with clinical or outcome data, thus providing novel imaging biomarkers [90]. Radiomics has been quite successful in oncology [91, 92]; however, the experience in cardiovascular imaging is limited. Recently, few studies demonstrated the feasibility and potential clinical value of radiomics analysis in cardiac CT and MRI images.

In a proof-of-concept study, Mannil et al. [93] tested radiomic features and six machine learning-based classifiers to detect myocardial infarction on unenhanced, low-radiation dose cardiac CT images of 57 patients with acute and chronic myocardial infarction and 30 controls. The authors were able to distinguish controls and patients with acute or chronic myocardial infarction with an AUC value of 0.78. This study was inspired by a previous investigation by Baessler et al. [94], who showed the potential of texture analysis in enabling the automatic diagnosis of small and large myocardial infarction on unenhanced cardiac cine MRI reaching AUC value of 0.92.

Similar studies by Larroza et al. [95] applied radiomics to discriminate nonviable, viable, and remote segments in unenhanced cine MRI scans of 50 patients. Among developed support vector machine classifiers, the best one showed AUC values on testing data equal to 0.935, 0.819, and 0.794 for nonviable, viable, and remote segments, respectively. The same group previously reported a radiomic analysis on late gadolinium cardiac MRI of 44 subjects that allowed to distinguish acute from chronic myocardial infarction with an AUC of 0.86 ± 0.06 (mean ± SD) during nested cross-validation [96].

Radiomics has also been reported to predict relevant clinical outcomes, such as post-myocardial infarction arrhythmias. Kotu et al. [97] investigated 34 patients with chronic myocardial infarction and performed a radiomic analysis of the myocardial scar. With this approach, they were able to identify patients with a high-risk of developing post-myocardial infarction fatal arrhythmias with an average AUC of 0.92 during a nested cross-validation.

These investigations (Table 1) are the foundational evidence that AI can be used in cardiovascular imaging to overcome the limits of a purely visual image assessment and provide a more complete evaluation, even in patients where late gadolinium enhancement imaging is not feasible. More AI-based applications are being developed in other areas of cardiovascular imaging; however, such applications go beyond the scope of this paper and have been extensively discussed [79, 90, 98–100].

**Table 1:** Artificial intelligence applications in ischemic heart disease

Authors (year) [reference]	AI approach	Imaging modality	Description
Zreik et al (2018) [85]	Deep learning	CT	Significant coronary stenosis detection based on left ventricular myocardium analysis
van Hamersvelt et al (2019) [86]	Deep learning	CT	Significant coronary stenosis detection based on left ventricular myocardium analysis in patients with intermediate stenosis
Xu et al (2017) [87]	Deep learning	MRI	Myocardial infarction size automatic detection
Zhang et al (2019) [88]	Deep learning	MRI	Chronic myocardial infarction diagnosis based on non-contrast cardiac cine MRI
Mannil et al (2018) [93]	Deep learning and radiomics	CT	Myocardial infarction detection based on non-contrast cardiac CT
Baessler et al (2018) [94]	Radiomics	MRI	Subacute and chronic myocardial infarction detection on non-contrast cardiac CT
Larroza et al (2018) [95]	Radiomics	MRI	Distinction between nonviable, viable, and remote segments on enhanced cine MRI
Larroza et al (2017) [96]	Radiomics	MRI	Acute and chronic myocardial infarction distinction based on cine and LGE cardiac MRI
Kotu et al (2015) [97]	Radiomics	MRI	Fatal arrhythmias risk assessment based on myocardial scar analysis from LGE cardiac MRI

*AI* Artificial intelligence, *CT* Computed tomography, *LGE* Late gadolinium enhancement, *MRI* Magnetic resonance imaging

Despite the promising initial results, there are concerns regarding the translation of AI-based tools into clinical practice. One of the main hurdles is the lack of a clear explanation of how these algorithms work. AI relies on machine learning and deep learning algorithms whose complexity makes them essentially a “black-box” system. The lack of transparency and explainability could result in limited trust and therefore, restricted use of AI in healthcare [101]. A similar effect could be caused by the input data used to train the algorithms, which should be as representative as possible of the population they are going to serve. The challenge of the next wave of AI will be to use more transparent algorithms and apply those algorithms to a larger scale, which most likely implies the collaboration of multiple centers around the world.

## Future outlook

As we reported in this review article, a variety of technological developments in cardiac imaging is expected to open new avenues in more advanced visualization, quantification, and prognostication of IHD. The potential new techniques, such as photon-counting CT, and low- and ultrahigh-field MRI, may bring major changes in our current clinical imaging practice. In combination with AI-based approaches, our imaging strategies, analysis approaches, and overall efficiency may see such substantial improvements that we have not seen for years in radiology.

## Data Availability

Not applicable

## Abbreviations

AI: Artificial intelligence
AUC: Area under the curve
CCTA: Coronary computed tomography angiography
CT: Computed tomography
CUDA: Coronary ultrafast Doppler angiography
DL: Deep learning
IHD: Ischemic heart disease
MRI: Magnetic resonance imaging
PET: Positron emission tomography
RGD: Arginyl-glycyl-aspartic acid
